# microRNA-211 regulates cell proliferation, apoptosis and migration/invasion in human osteosarcoma via targeting EZRIN

**DOI:** 10.1186/s11658-019-0173-x

**Published:** 2019-07-09

**Authors:** Yihua Pei, Qin Yao, Yingchao Li, Xin Zhang, Bozhen Xie

**Affiliations:** 1 Central laboratory, ZhongShan Hospital XiaMen University, Xiamen, 361004 China; 2 Fujian Provincial Key Laboratory of Chronic Liver Disease and Hepatocellular Carcinoma (Xiamen University Affiliated ZhongShan Hospital), Xiamen, 361004 China; 3 Department of Spine Surgery, ZhongShan Hospital XiaMen University, No. 201 Hubin South Road, Xiamen, 361004 China; 4 Department of Rehabilitation, ZhongShan Hospital XiaMen University, Xiamen, 361004 China

**Keywords:** miR211, EZRIN, Osteosarcoma, Cell viability, Cell apoptosis

## Abstract

**Background:**

In recent years, microRNA-211 (miR211) has been considered as a tumor suppressor in multiple malignancies. However, the function of miR211 in human osteosarcoma has not been explored intensively so far. In this study, the relationship between miR211 and EZRIN was analyzed in human osteosarcoma.

**Methods:**

The expression levels of miR211 and EZRIN were measured in both human osteosarcoma cells and tissues. The direct regulatory relationship between miR211 and EZRIN was evaluated using dual-luciferase assay. The effect of miR211 and EZRIN overexpression on cell proliferation, migration/invasion, and apoptosis was detected.

**Results:**

The expression of miR211 was obviously lower in osteosarcoma tissues than paracancerous tissues. EZRIN was identified as the direct target of miR211, and up-regulation of miR211 increased the percentage of cell apoptosis, and suppressed cell proliferation as well as cell migration/invasion via directly regulating EZRIN.

**Conclusions:**

Our study indicated that miR211 has an important role in the development and progress of osteosarcoma, and it might become a novel target in the diagnosis and treatment of human osteosarcoma.

**Electronic supplementary material:**

The online version of this article (10.1186/s11658-019-0173-x) contains supplementary material, which is available to authorized users.

## Background

Osteosarcoma is the most prevalent primary malignant bone cancer in the word, and this disease can affect the growth of bones seriously if it happens in young people [1, 2]. In recent years, the incidence of osteosarcoma has been increasing by about 1.4% each year worldwide, and the early prognosis of osteosarcoma is still poor. [3, 4]. One study indicated that only about 50% of patients could survive in the following 5 years [5]. Therefore, the development of more effective therapeutic methods and novel prognostic molecular markers is necessary to improve the patient survival rate.

Many microRNAs (miRNAs) have been demonstrated to act as oncogenes or tumor suppressor genes. During the process of cancer development and progression, miRNAs were indicated to have a significant role in regulation. In addition, those miRNAs also can be used as novel molecular biomarkers for cancer prognosis, even cancer targeted therapies [6–9]. Recently, some studies indicated that microRNA-211 (miR211) suppressed the metastatic and invasive ability of different types of cancers (e.g. cervical cancer, breast cancer, and melanoma) [10–14]. However, the detailed function of miR211 in human osteosarcoma has not been explored so far.

EZRIN, a cytoskeleton-associated protein, is a member of the ezrin-radixin-moesin (ERM) family [15]. EZRIN can participate in cell migration/invasion, as well as cell proliferation [16, 17]. The regulating function of EZRIN in malignant behavior has been reported in several studies [18–21]. More importantly, EZRIN also has been demonstrated to correlate with inferior outcome in several types of cancers [16, 21–23]. For patients with colorectal cancer, high expression of EZRIN usually can predict a poor survival rate [24], and some researchers showed that silencing of EZRIN could inhibit the metastasis of breast cancer cells [19]. In addition, some meta-analysis results indicated that EZRIN positive immunoexpression could confer worse survival as well as a higher risk of recurrence in osteosarcoma patients [25]. Of course, the clinical significance of EZRIN needs to be further confirmed by large prospective studies. Recently, one group reported the regulating relationship between miR-211-5p and EZRIN in tongue squamous cell carcinoma. The researchers found that miR-211-5p could bind to the 3′-UTR of EZRIN mRNA and miR-211-5p upregulation significantly impaired tongue squamous cell carcinoma proliferation and resumed the chemo-sensitivity [26]. However, whether such regulation still holds any function or clinical significance in osteosarcoma is unclear.

In the present study, we mainly validated EZRIN as the direct target gene of miR211 in human osteosarcoma cells. We found that miR211 overexpression could induce osteosarcoma cell apoptosis and suppress osteosarcoma cell proliferation, as well as migration/invasion, indicating that miR211 holds the potential to become a novel diagnosis marker and therapy target for human osteosarcoma.

## Methods

### Osteosarcoma samples

This study was approved by the Committee on the Ethics of Human Subject Research of ZhongShan Hospital XiaMen University. In this study, 4 patients donated their osteosarcoma tissues and paracancerous tissues for our research. All volunteers who donated tissues have provided their written informed consent. The ethics committee in ZhongShan Hospital XiaMen University has approved this consent procedure.

### Cell culture

Human osteosarcoma cell line 143B was purchased from the American Type Culture Collection (ATCC). The cells were cultured using Dulbecco’s Modified Eagle Medium (Hyclone) supplemented with 10% Fetal Bovine Serum (Gibco), 0.1 g/mL streptomycin and 100 U/mL penicillin (Sigma) in a humidified 37 °C incubator with 5% CO_2_. The culture medium was changed every 2 days, and the cells were passaged by 1:4 dilution every 5–6 days.

### miR211 and pCI-EZRIN preparation

miR211 (5′-UUCCCUUUGUCAUCCUUCGCCU-3′) and miR204 (5′-UUCCCUUUGUCAUCCUAUGCCU-3′) were synthesized by GenePharma (Shanghai, China). The control miRNA (5′-AAGGGAAACAGUAGGAAGCGGA-3′) was used as a negative control (ctrl miRNA). The siRNA targeting miRNA-211 (5′-AGGCGAAGGAUGACAAAGGGAA-3′) was applied as a miR211 inhibitor. The siRNA targeting miR204 (5′-AGGCGAUAGGAUGACAAAGGGAA-3′) was applied as a miR204 inhibitor and the control siRNA (5′-UUCUCCGAACGUGUCACGU-3′) was used as the negative control (ctrl siRNA). To generate EZRIN overexpressing vectors, the *Homo sapiens* EZRIN-coding sequences plus 3′-UTR were obtained by reverse transcription PCR and further cloned into a pCI-based retroviral plasmid (Addgene). Human EZRIN siRNA was purchased from Santa Cruz Biotechnology (sc-35,349).

Cell transfection was performed using Lipofectamine 3000 transfection reagent (Invitrogen) according to the manufacturer’s instructions.

### Real-time quantitative PCR (qPCR)

The operation of qPCR was the same as in our previous study [27]. The primer sequences (5′-3′) used in qPCR are as follows:

- miR211 Forward (F): 5′- ACACTCCAGCTGGGTTCCCTTTGTCATCCT -3′
- Reverse (R): 5′-CTCAACTGGTGTCGTGGAGTCGGCAATTCAGTTGAGAGGCGAAG-3′
- miR204 Forward (F): 5′- ACACTCCAGCTGGGTTCCCTTTGTCATCCT -3′
- Reverse (R): 5′-CTCAACTGGTGTCGTGGAGTCGGCAATTCAGTTGAGAGGCATAG -3′
- U6
  - Forward (F): 5′-CTCGCTTCGGCAGCACA-3′
  - Reverse (R): 5′-AACGCTTCACGAATTTGCGT-3′
- EZRIN
  - Forward (F): 5′-AGCACACGGAGCACTGCAGG-3′
  - Reverse (R): 5′-GTAACTCGGACATTGATTGG-3′
- 18srRNA
  - Forward (F): 5′-CCTGGATACCGCAGCTAGGA-3′
  - Reverse (R): 5′-GCGGCGCAATACGAATGCCCC-3′

### Western-blot

The cell samples were harvested with RIPA lysis buffer, and the protein content of cell lysates in different groups was further detected with the BCA protein estimation kit (Pierce, USA). The performance of western blot was as described in the cited literature [28–30]. The primary antibodies used in the study were anti-EZRIN (1:1000; Abcam) and anti-GAPDH (1:1000; Abcam). Anti-mouse or rabbit secondary antibody (HRP; 1:5000; Santa) and an Amersham ECL kit (GE Healthcare) were further used to detect the protein.

### Dual luciferase assay

The 3′-UTR sequences of EZRIN were obtained by PCR and further cloned into psiCHECK-based luciferase plasmid (Addgene). The mutated psiCHECK-EZRIN 3′-UTR was induced as the reference [31, 32].

### Cell proliferation and cell cycle analysis

To evaluate cell proliferation ability, the proliferation index of each group was detected with the CCK-8 method (Dojindo) as the reference [33–35]. Cell cycle was analyzed using PI staining as in our previous study [27].

### Cell apoptosis assay

In our study, cell apoptosis assay was performed using the Cell Apoptosis Assay Kit (Life Technologies) according to the manufacturer’s instructions as in our previous study [27].

### Cell migration and invasion assay

In our study, the migration and invasion of cancer cells were measured with Transwell plates (8 μm pore filter, Corning Costar) as in the cited literature [36, 37].

### Statistical analysis

**Fig. 1:**
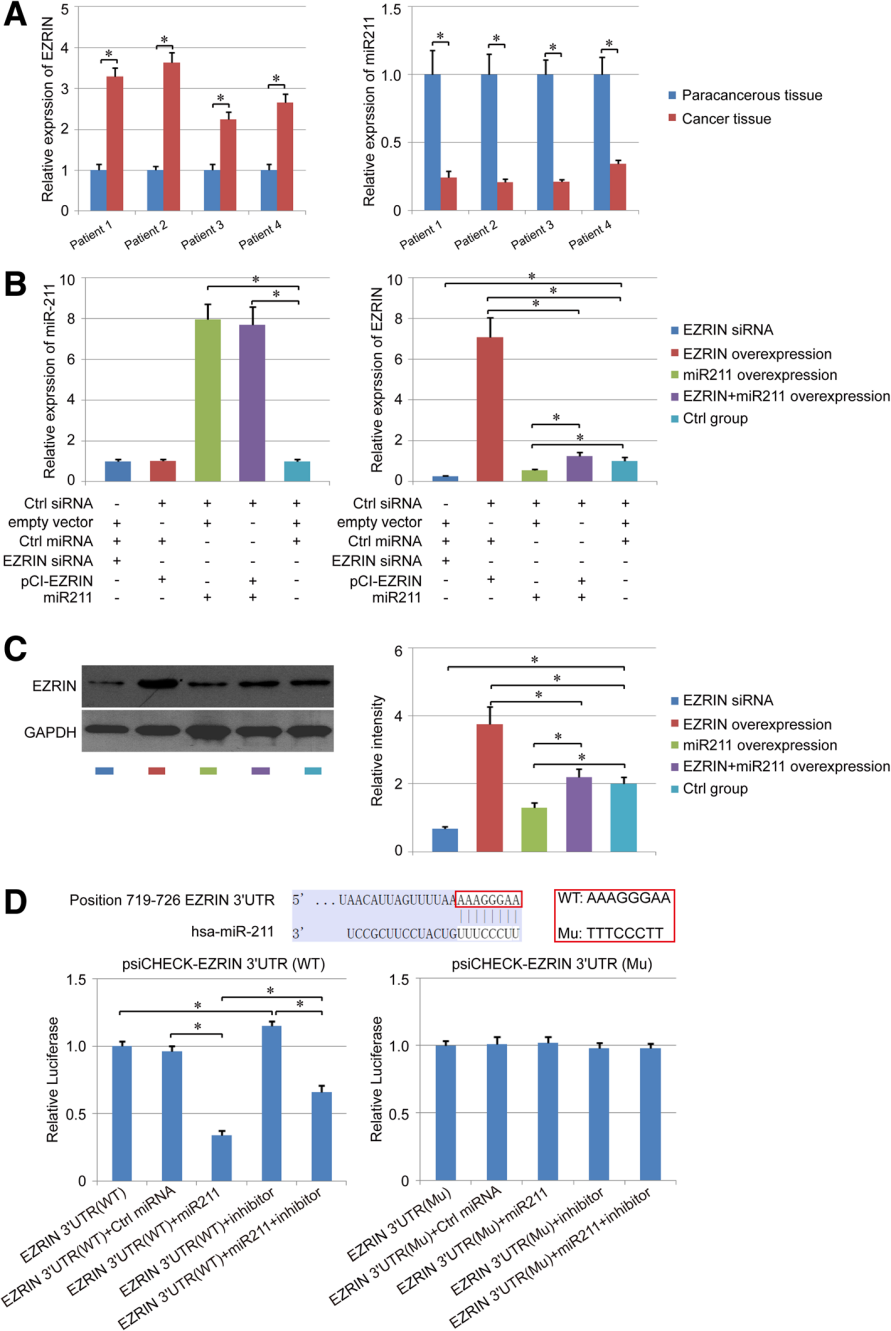
The expression of EZRIN was regulated by miR211 directly. **a**. the expression of EZRIN and miR211 was respectively measured by qPCR in human osteosarcoma tissues and paracancerous tissues. **b**-**c**. miR211 overexpression inhibited the expression of EZRIN in human osteosarcoma cells. The level of miR211 in different groups was detected with qPCR first (**b**). Then, the expression of EZRIN was evaluated with qPCR (**b**) and immunoblot (**c**) respectively. **d**. The direct binding relationship between miR211 and EZRIN. The targeted modulation between EZRIN and miR211 was analyzed using the dual-luciferase system. *: *P* < 0.05 between the two groups

**Fig. 2:**
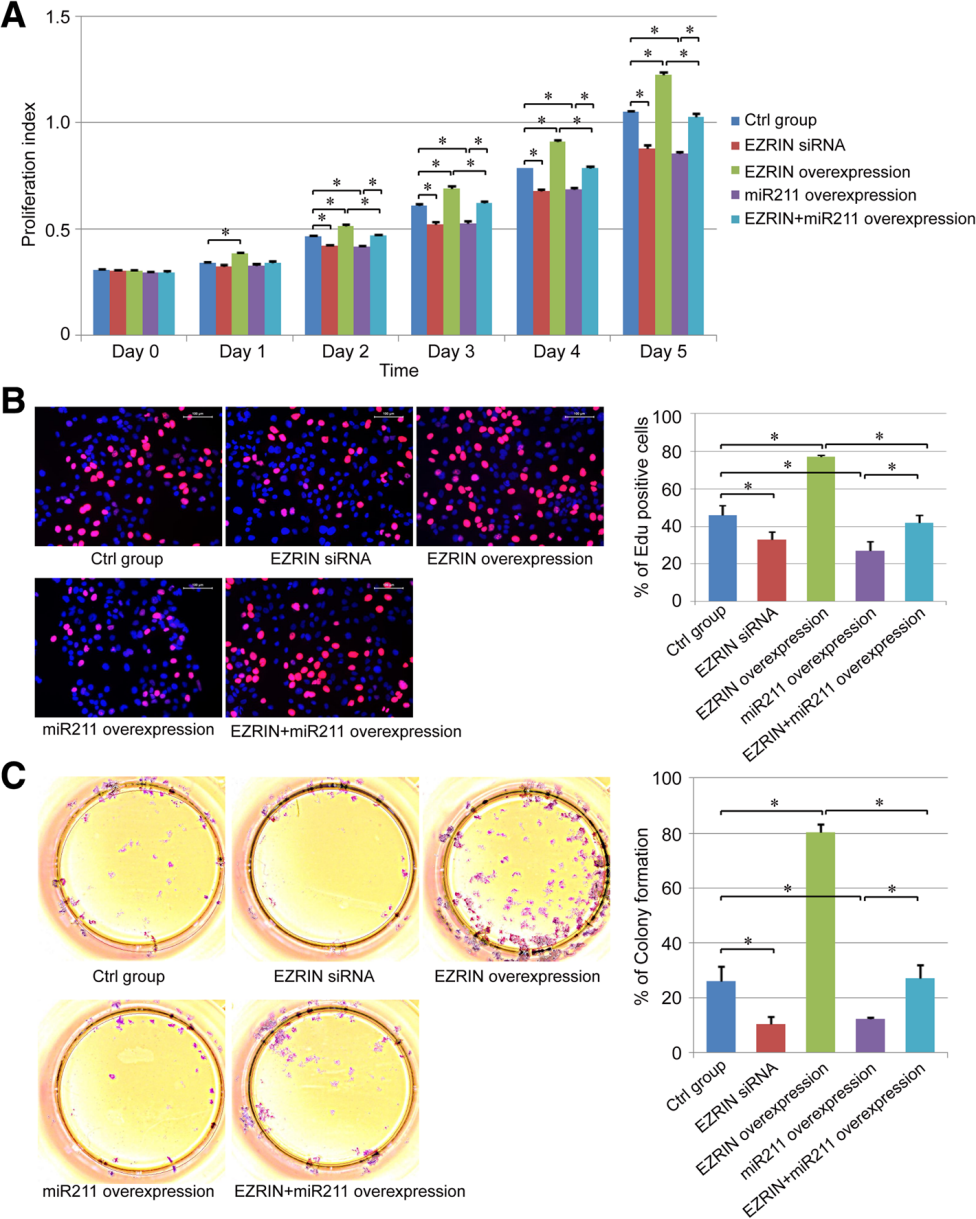
miR211 overexpression inhibited the proliferation ability of human osteosarcoma cells. **a**-**c**. The effect of miR211 and EZRIN on cell proliferation was detected with CCK-8 (**a**), EdU staining (**b**), and colony formation assay (**c**). *: *P* < 0.05 between the two groups

In this study, the results were expressed as means ± SEM, and statistical assay was performed using SPSS 17.0 software. Unpaired Student’s t-tests were used to analyze the means of two groups. One-way ANOVA with Bonferroni’s correction was used to analyze the means of three or more groups. P<0.05 was considered significant statistically. In Fig. 1, the level of paracancerous tissue group was regarded as “1”. In Fig. 2, The level of the control group (EZRIN-3′-UTR(WT) and EZRIN-3′-UTR(Mu)) was regarded as “1”.

## Results

### EZRIN is the direct target of miR211 in human osteosarcoma cells

Human osteosarcoma tissues and paracancerous tissues (*n* = 4) were harvested to evaluate the relationship between miR211 and EZRIN using qPCR first. The result showed that the mRNA level of EZRIN was much higher in human osteosarcoma tissues than human paracancerous tissues (*P*<0.05), while the level of miR211 showed an opposite tendency in human osteosarcoma tissues and paracancerous tissues (Fig. 1a), suggesting that there might be a negative regulatory relationship between miR211 and EZRIN in the patients’ osteosarcoma tissues.

In addition, a human osteosarcoma cell line, 143B, was used to analyze the negative regulatory relationship between miR211 and EZRIN in osteosarcoma. We found that both the mRNA and the protein level of EZRIN were inhibited by the up-regulation of miR211 in human osteosarcoma cells (*P*<0.05, Fig. 1b-c). In addition, compared with the EZRIN overexpression group, 143B co-transfected with miR211 and pCI-EZRIN showed a much lower level of EZRIN (*P*<0.05, Fig. 1b-c). We further detected the subcellular localization of EZRIN in each group, and the results showed that the EZRIN subcellular localization was similar to each other in different groups, and all of the positive staining was located in the cytoplasm (Additional file 1: Figure S1).

The potential target genes of miR211 were analyzed using TargetScan, indicating that miR211 can target the 3′-UTR of EZRIN and regulate this gene directly (the binding relationship is shown in Fig. 1d). Therefore, both wild-type (WT) and mutant-type (Mu) 3′-UTR of EZRIN were cloned into the psi-CHECK vector, followed by 143B cell transfection and dual-luciferase assay. We found that miR211 repressed the luciferase activity in the WT EZRIN-3′-UTR group significantly, but did not show an obvious effect in the Mu EZRIN-3′-UTR group (*P*<0.05, Fig. 1d). Additionally, the inhibitory effect of miR211 was shown by the transfection with miR211 inhibitor in the WT EZRIN-3′-UTR group, but not in Mu EZRIN-3′-UTR group (*P*<0.05, Fig. 1d). Taken together, these assays revealed the direct targeted regulation between EZRIN and miR211.

### Effect of miR211 on the proliferation ability of osteosarcoma cells

The CCK-8 results showed that osteosarcoma cell proliferation ability was inhibited by the EZRIN siRNA, while the proliferation index could be enhanced via EZRIN overexpression. In addition, the overexpression of miR211 suppressed osteosarcoma cell proliferation ability obviously and also inhibited the promoting effect of EZRIN overexpression (Fig. 2a).

In addition, the proliferation ability of different group was further detected via EdU assay and colony formation assay. The results showed that both the colony formation ability and the positive staining of EdU could be rescued with the transfection of EZRIN siRNA, and increased in the EZRIN overexpression group. miR211 transfection also decreased the EdU staining and the number of colonies in normal osteosarcoma cells, and EZRIN overexpressed 143B cells, which was consistent with the CCK-8 results (Fig. 2b-c).

**Fig. 3:**
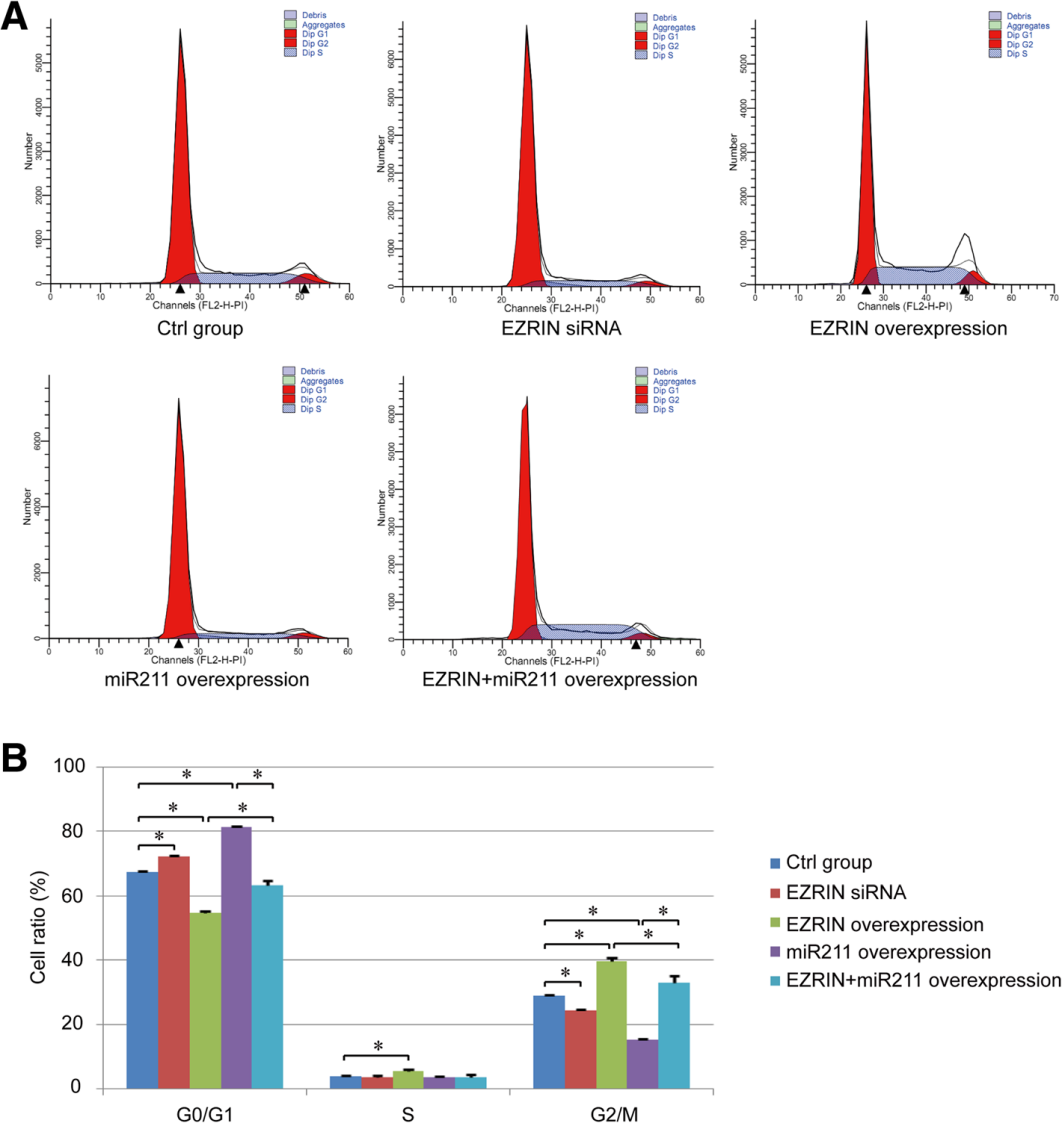
Effect of miR211 and EZRIN on cell cycle of osteosarcoma cells. **a**. Cell cycle assay of different groups. **b**. The summary of percentage of G0/G1, S and G2/M phase in different groups. *: *P* < 0.05 between the two groups

The regulatory effect of miR211 and EZRIN on the cell cycle was further measured via flow cytometry assay. We found that both miR211 overexpression and EZRIN siRNA transfection increased the percentage of G0/G1 phase and decreased the percentage of S phase in the human osteosarcoma cells. The opposite phenomenon could be found in the EZRIN overexpression group, which could be rescued by the up-regulation of miR211 to some degree (*P*<0.05, Fig. 3a-b). Therefore, miR211 could suppress the osteosarcoma cell proliferation ability via the targeted regulation of EZRIN.

### Effect of miR211 on osteosarcoma cell apoptosis

**Fig. 4:**
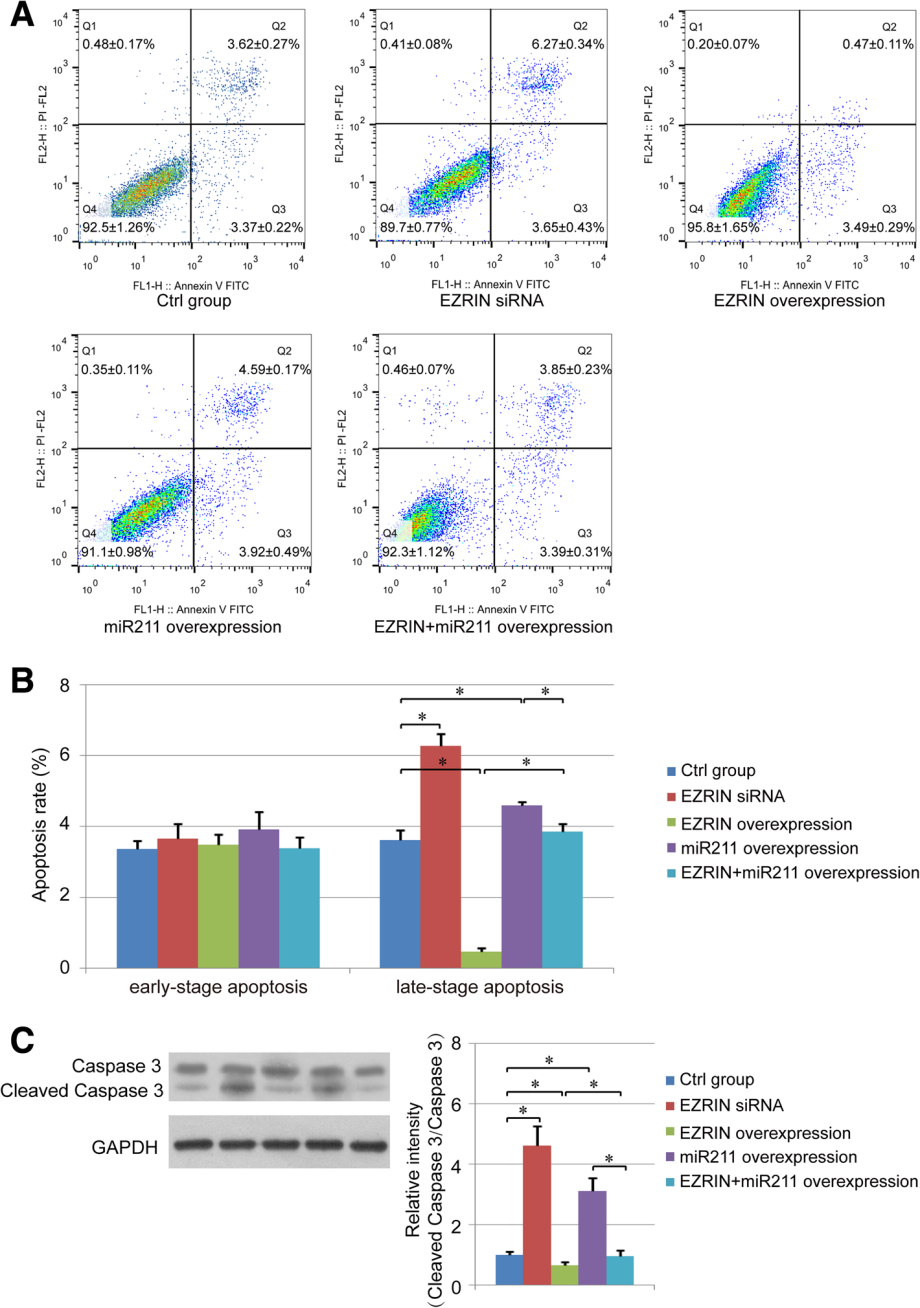
Effect of miR211 and EZRIN on cell apoptosis. **a**. Cell apoptosis was analyzed using Annexin V-FITC/PI staining. **b**. The summary of apoptotic rate (%) of early (lower right quadrants) and late (upper right quadrants) stage apoptotic cells. **c**. western blot detection of whole caspase 3 and cleaved caspase 3. *: *P* < 0.05 between the two groups

In our study, cell apoptosis was detected using Annexin V/PI staining. Both early-stage apoptotic cells (Annexin V-positive and PI-negative) and late-stage apoptotic cells (Annexin V-positive and PI-positive) were analyzed herein, but only the percentage of late-stage apoptotic cells showed some difference among the groups. We found that the percentage of late-stage apoptotic cells was increased in both the EZRIN siRNA group and the miR211 overexpression group, while it decreased in the EZRIN overexpression group compared with the control group. Also, in the EZRIN+miR211 group there were fewer apoptotic cells compared with the single miR211 overexpression group (Fig. 4a-b). In addition, the level of whole caspase 3 and cleaved caspase 3 were detected herein, and the results indicated that the amount of cleaved caspase 3 was decreased in the EZRIN overexpression group, and increased in the EZRIN siRNA group and miR211 overexpression group, which was consistent with the Annexin V/PI staining results (Fig. 4c).

### miR211 inhibits osteosarcoma cell migration and invasion

**Fig. 5:**
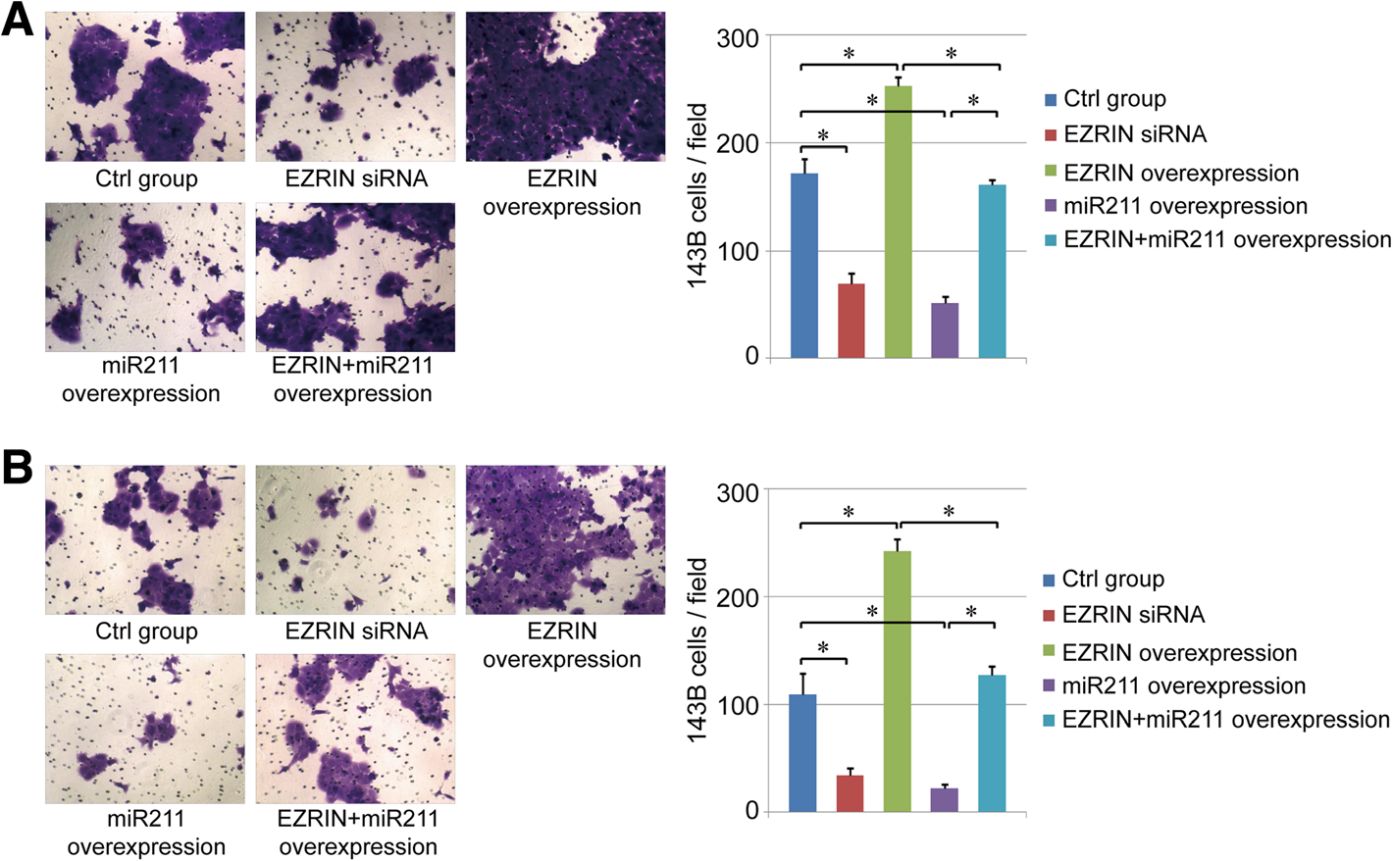
Cell migration and invasion assay. **a**-**b**. Effect of miR211 and EZRIN on the migration (**a**) and invasion (**b**) ability of human osteosarcoma cells. *: *P* < 0.05 between the two groups

The effect of EZRIN and miR211 on cell migration and invasion ability was further analyzed in this study. Our results indicated that both cell migration and invasion could be enhanced through EZRIN overexpression. Compared with the control group, the migration and invasion ability of human osteosarcoma cells were suppressed in the EZRIN siRNA group and miR211 overexpression group. In addition, miR211 overexpression can also inhibit the influence of EZRIN overexpression on both migration and invasion ability of 143B cells, suggesting the key position of miR211 in migration/invasion of human osteosarcoma cells (*P*<0.05, Fig. 5a-b).

## Discussion

Some studies have demonstrated that miR211 holds a significant position in the development and progression of cancers, and even other diseases [10–12, 38]. Recently, some researchers found that miR211 inhibited invasion and epithelial-to-mesenchymal transition of cervical cancer cells via targeting MUC4 [10]. Moreover, miR211 was indicated to inhibit the proliferation, migration, and invasion of thyroid tumor cells through downregulating SOX11 [12]. Additionally, some researchers showed that miR211 could inhibit cortical neuron differentiation and survival, which might contribute to the synaptic failure, cognitive dysfunction and neuronal loss in Alzheimer’s disease [38]. However, the relationship between miR211 and human osteosarcoma has not been explored so far. In the present study, we mainly demonstrated that miR211 targeted the 3′-UTR of EZRIN, and regulated the expression of EZRIN negatively in both human osteosarcoma tissues and cells. The miR211 overexpression promoted cell apoptosis and inhibited the proliferation and migration/invasion ability of osteosarcomas cell significantly, indicating the key role of miR211 in the development and progress of human osteosarcoma.

However, the detailed mechanism of the regulatory function of miR211 in human osteosarcoma is still far away from our understanding. Why the development of osteosarcoma is accompanied by down-regulation of miR211 still needs further investigation. One study indicated that the level of hypermethylation in the promoter of some miRNAs could be enhanced in some cancer cells, resulting in the down-regulation of those miRNAs [39]. Therefore, the hypermethylation of miRNA promoter may hold an important position in the regulation of miRNA in the development and progression of cancers. However, whether such a hypermethylation mechanism is still applicable for the regulation of miR211 still requires our further intensive investigation. In addition, four patient samples and only one osteosarcoma cell line were involved in our research. It is hard to perform linear regression analysis of miR211, EZRIN and osteosarcoma development. Therefore, more patient samples as well as osteosarcoma cell lines are necessary for our further investigation.

Metastasis is considered a hallmark of cancer, as well as the leading cause of mortality among patients with cancer. Thus, cancer is not only a disease of uncontrolled cell growth, but also a disease of uncontrolled cell migration/invasion. The development of novel strategies against cancer cell migration/invasion is necessary to improve the patient survival rate. Several groups have demonstrated that miR211 could inhibit cell migration/invasion ability via targeting different genes, including STAT5A [40], SPARC [41], FABP4 [42], SETBP1 [11], and CDC25B [43]. Our results indicated that miR211 affects cell proliferation and migration/invasion ability via regulating EZRIN. However, in the trans-well assay, cell proliferation might have influenced the assay results, because of the different cell number in different groups. Our results indicated that EZRIN overexpression increased proliferation ability by only about 20%, but promoted migration and invasion ability by 50–100%, indicating that EZRIN and miR211 might regulate the migration and invasion in a proliferation-independent manner. Of course, further investigations are needed to confirm this conclusion.

MiR204 and miR211 share a similar seed site sequence, and many genes are targeted by miR204 and miR211 at the same time. However, the functions of such two miRNAs are still controversial [44–46]. In osteosarcoma, miR204 has been indicated to suppress the cell viability via targeting EBF2 [47]. In our study, we tried to analyze the role of miR211 in osteosarcoma. To evaluate the specificity of primers and inhibitor in our study, we tried to detect the overexpression effect using both the miR204 primer and the miR211 primer, and the results indicated that only the miR211 primer worked in the qPCR detection of cells transfected with miR211, but not for the miR204 primer. In addition, it seems that the basal level of miR211 was much higher than that of miR204 in the 143B cells. After the transfection with miR211 inhibitor, only miR-211 showed down-regulation, and the level of miR-204 did not change obviously, indicating that miR-211 inhibitor is still specific in our study (Additional file 2: Figure S2). Furthermore, a study showed that the analysis of the two host genes (TRPM3 for miR204 and TRPM1 for miR211) could determine the contribution of different miRNAs effectively [48], which helped us analyze the position of miR204 and miR211 more accurately.

In addition, some researchers have found other genes which could be regulated by miR211 directly. For example, Chen et al. found that miR211 was significantly downregulated in triple-negative breast cancer, and SETBP1 was further identified as a target of miR211. The overexpression of miR211 inhibited the expression of SETBP1 significantly and the restoration of SETBP1 expression also could reverse the inhibitory effect of miR211 on cancer cell proliferation as well as metastasis [11]. Therefore, miR211 can regulate the viability of cancer cells through different pathways. Perhaps more attention should be paid to the analysis of different signal pathways associated with miR211. A better understanding of the miR211 regulatory mechanism in different cancers and which gene plays the most important role during the process of miR211 regulation can help us develop more reliable and effective methods to treat patients with cancer.

Our previous study had indicated that miR96 acts as a tumor suppressor gene in human osteosarcoma via target regulation of EZRIN. Potentially the combination of the two miRNAs (miR96 and miR211) might show better regulation of EZRIN, because the target sites for different miRNAs are different. We also performed co-transfection of miR-96 and miR-211 in osteosarcoma cells and detected the cell proliferation ability, and the results showed that the combination of the two miRNAs exhibits a stronger inhibitory effect on cell proliferation than single transfection (Additional file 3: Figure S3). Therefore, the combination of different miRNAs might provide a novel insight for pre-clinical study of cancer treatment.

## Conclusion

In conclusion, we mainly found that EZRIN can be targeted by miR211 in human osteosarcoma, and miR211 regulated the expression of EZRIN negatively. miR211 overexpression increased the level of apoptosis in a human osteosarcoma cell line and inhibited cell proliferation ability as well as cell migration/invasion ability in 143B cells via suppressing the expression of EZRIN. Overall, our results indicated that miR211 hold the potential to become a novel diagnosis marker in human osteosarcoma, as well as a target for gene therapy.

## Additional files

Additional file 1: **Figure S1.** Immunofluorescence staining of EZRIN in different groups. Scale bar = 50 μm. (TIF 6845 kb)

Additional file 2 : **Figure S2.** Evaluation of the specificity of miR211 primers and inhibitor in our study. A. Comparison of miR211 primer and miR204 primer in qPCR detection. To induce miR211 overexpression, the cells were transfected with miR211, then harvested for qPCR detection using different primers. B. Evaluation of the specificity of miR211 inhibitor using qPCR. To induce miR211 down-regulation, the cells were transfected with miR211 inhibitor, then harvested for qPCR detection using different primers. The level of ctrl group detected using miR211 primer was considered as “1”. *: *P* < 0.05 between the two groups. (TIF 754 kb)

Additional file 3: **Figure S3.** Effect of miR211 combined with miR96 on cell proliferation. *: *P* < 0.05 between the two groups. (TIF 620 kb)

## Data Availability

All data generated or analyzed during this study are included in this manuscript.

## Abbreviations

ATCC: American Type Culture Collection
ERM: Ezrin-radixin-moesin
miR211: microRNA-211
miRNAs: microRNAs
Mu: Mutant-type
qPCR: Real-time quantitative PCR
WT: Wild-type
