# Supplementary figures and images for: microRNA-211 regulates cell proliferation, apoptosis and migration/invasion in human osteosarcoma via targeting EZRIN
Source: Cell Mol Biol Lett. 2019 Jul 9;24:48. doi: 10.1186/s11658-019-0173-x (PMC6617937; doi:10.1186/s11658-019-0173-x)

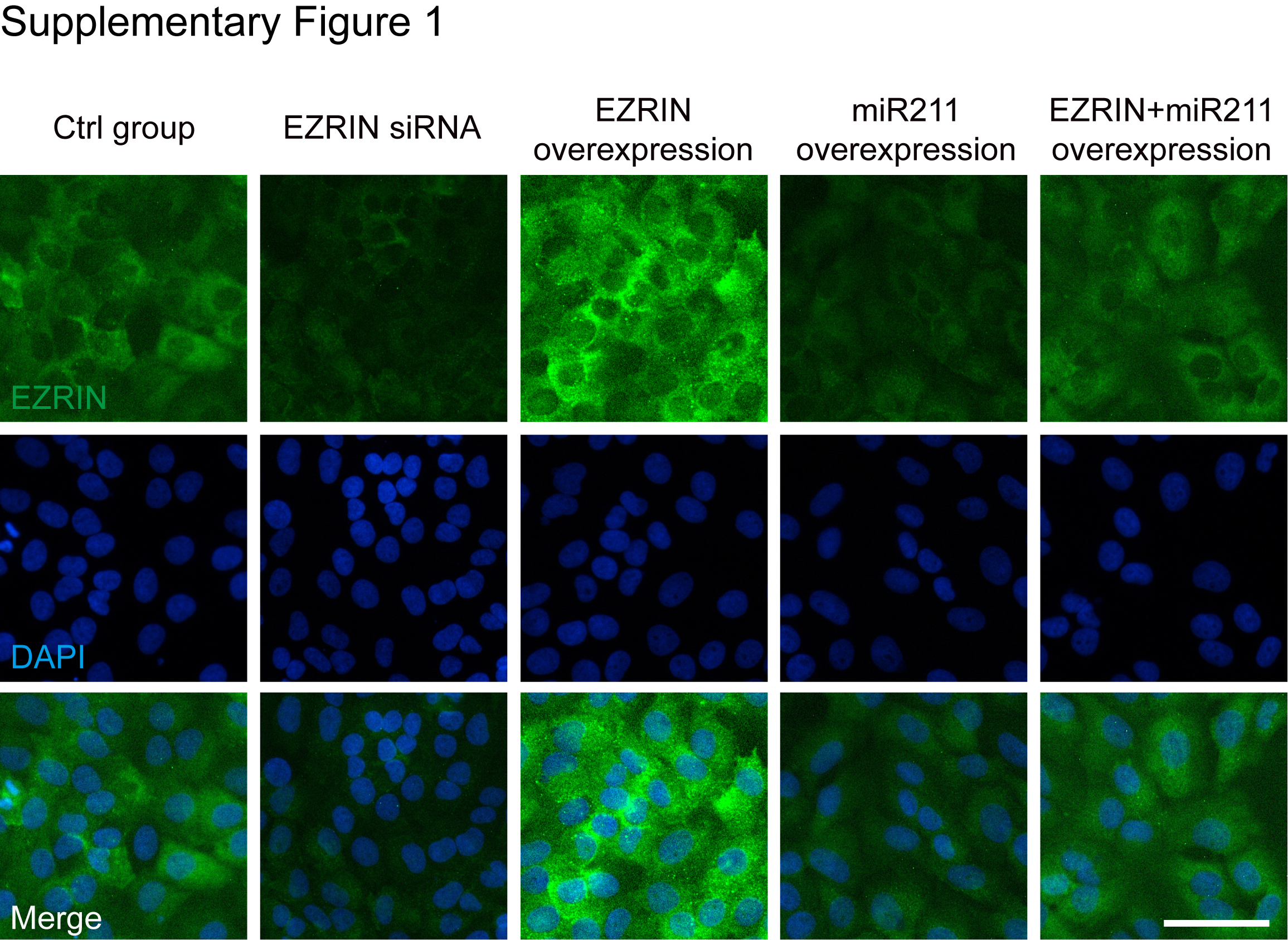

Supplement: Supplementary file 1 — Figure S1. Immunofluorescence staining of EZRIN in different groups. Scale bar = 50 μm. (TIF 6845 kb) [file 11658_2019_173_MOESM1_ESM.tif]

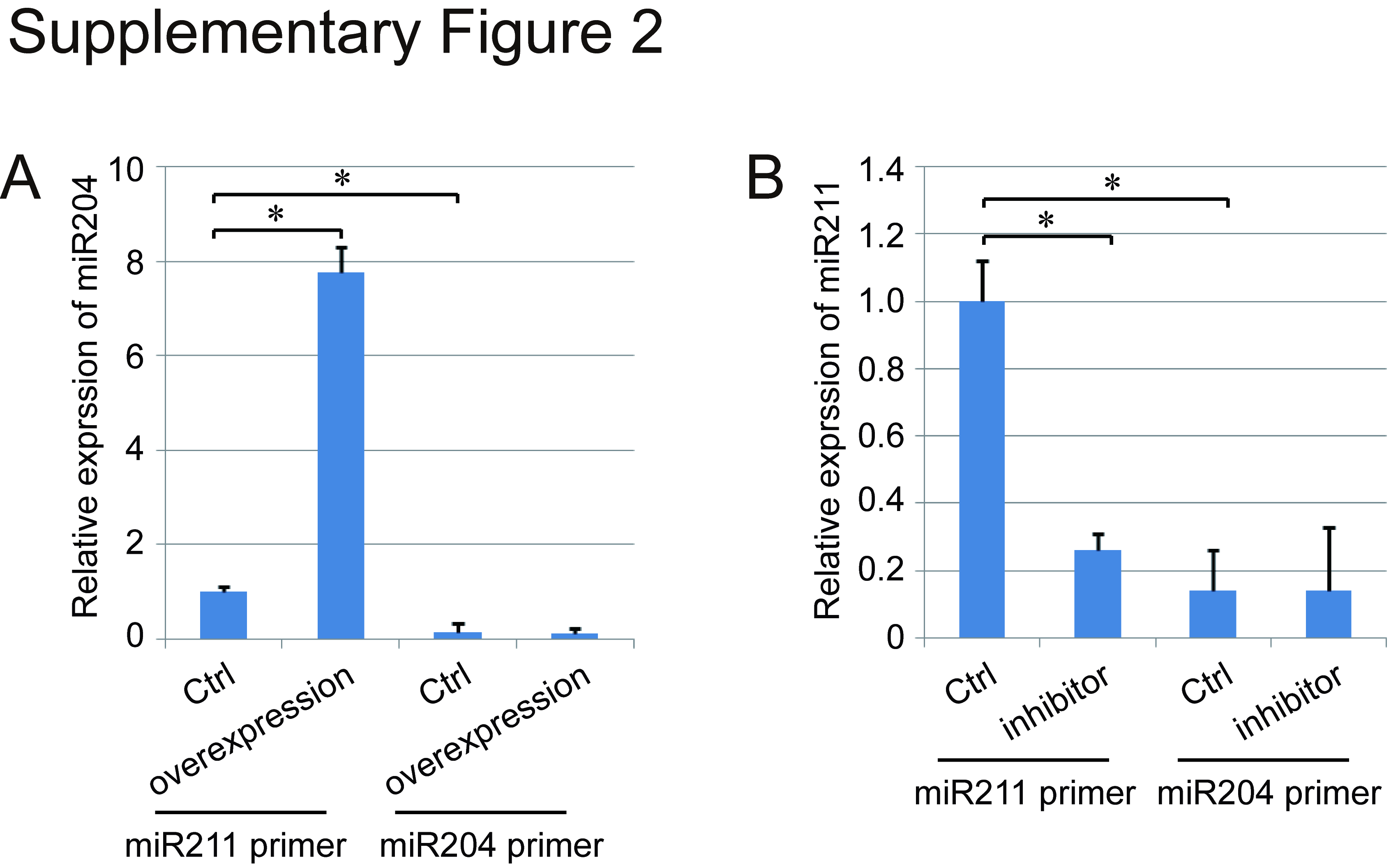

Supplement: Supplementary file 2 — : Figure S2. Evaluation of the specificity of miR211 primers and inhibitor in our study. A. Comparison of miR211 primer and miR204 primer in qPCR detection. To induce miR211 overexpression, the cells were transfected with miR211, then harvested for qPCR detection using different primers. B. Evaluation of the specificity of miR211 inhibitor using qPCR. To induce miR211 down-regulation, the cells were transfected with miR211 inhibitor, then harvested for qPCR detection using different primers. The level of ctrl group detected using miR211 primer was considered as “1”. *: P < 0.05 between the two groups. (TIF 754 kb) [file 11658_2019_173_MOESM2_ESM.tif]

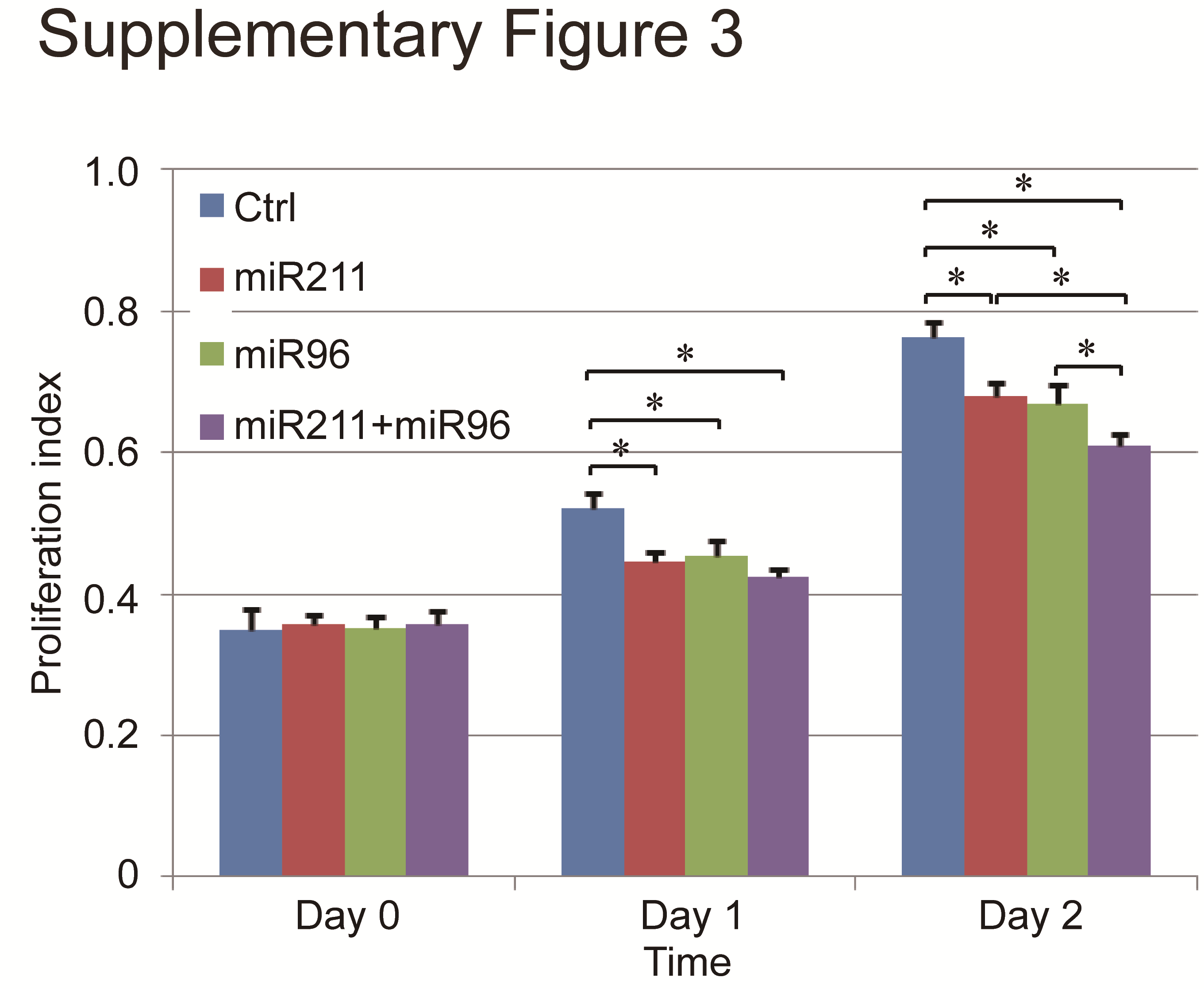

Supplement: Supplementary file 3 — Figure S3. Effect of miR211 combined with miR96 on cell proliferation. *: P < 0.05 between the two groups. (TIF 620 kb) [file 11658_2019_173_MOESM3_ESM.tif]
